# Voltammetric Determination of Homocysteine Using Multiwall Carbon Nanotube Paste Electrode in the Presence of Chlorpromazine as a Mediator

**DOI:** 10.1155/2012/902184

**Published:** 2012-05-22

**Authors:** Fathali Gholami-Orimi, Farshad Taleshi, Pourya Biparva, Hassan Karimi-Maleh, Hadi Beitollahi, Hamid R. Ebrahimi, Mohamad Shamshiri, Hasan Bagheri, Masoud Fouladgar, Ali Taherkhani

**Affiliations:** ^1^Department of Chemisty, Qaemshahr Branch, Islamic Azad University, Qaemshahr, Iran; ^2^Department of Applied Science, Qaemshahr Branch, Islamic Azad University, Qaemshahr, Iran; ^3^Department of Nanotechnology, Langaroud Branch, Islamic Azad University, Langaroud, Iran; ^4^Department of Chemistry, Science and Research Branch, Islamic Azad University, Mazandaran, Iran; ^5^Environment Department, Research Institute of Environmental Sciences, International Center for Science, High Technology & Environmental Sciences, Kerman, Iran; ^6^Department of Chemistry, Majlesi Branch, Islamic Azad University, Isfahan, Iran; ^7^Department of Medical Chemistry, School of Pharmacy and Pharmaceutical Sciences, Isfahan University of Medical Sciences, Isfahan, Iran; ^8^Department of Chemisty, Takestan Branch, Islamic Azad University, Takestan, Iran; ^9^Department of Biochemistry, Falavarjan Branch, Islamic Azad University, Falavarjan, Iran; ^10^Department of Physics, Takestan Branch, Islamic Azad University, Takestan, Iran

## Abstract

We propose chlorpromazine (CHP) as a new mediator for the rapid, sensitive, and highly selective voltammetric determination of homocysteine (Hcy) using multiwall carbon nanotube paste electrode (MWCNTPE). The experimental results showed that the carbon nanotube paste electrode has a highly electrocatalytic activity for the oxidation of Hcy in the presence of CHP as a mediator. Cyclic voltammetry, double potential step chronoamperometry, and square wave voltammetry (SWV) are used to investigate the suitability of CHP at the surface of MWCNTPE as a mediator for the electrocatalytic oxidation of Hcy in aqueous solutions. The kinetic parameters of the system, including electron transfer coefficient, and catalytic rate constant were also determined using the electrochemical approaches. In addition, SWV was used for quantitative analysis. SWV showed wide linear dynamic range (0.1–210.0 **μ**M Hcy) with a detection limit of 0.08 **μ**M Hcy. Finally, this method was also examined as a selective, simple, and precise electrochemical sensor for the determination of Hcy in real samples.

## 1. Introduction

Homocysteine was discovered by serendipity in 1934 as a byproduct of the digestion of methionine with hydriodic acid, a procedure used then for the determination of protein methionine [1]. The determination of Hcy has gained high interest within the biomedical community over recent years as it is a major biomarker for a wide range of diseases [2]. The distribution of Hcy among different tissues, cells, and intracellular compartments is an important factor affecting many physiological concentrations. In biological systems, Hcy is usually bound in a disulfide linkage; the usual level of the free unbound Hcy species is approximately 1-2% of the total Hcy concentration [3]. However, in patients with genetic disorders of Hcy metabolism or patients suffering from cardiovascular diseases, the concentration of free Hcy increases [4] and, therefore, its monitoring can be crucial to the medical community as a cardiac marker. Several methods have been proposed for the determination of Hcy that include gas chromatography-mass spectroscopy (GC/MS) [5], HPLC (or capillary electrophoresis) with fluorescent [6], laser-induced fluorescent [7], mass spectrometric [8], and electrochemical methods [9–11]. Electrochemical methods are known to possess such advantages as simplicity, high sensitivity, and ease in automation. Electrochemistry of Hcy revealed a slow behavior at solid electrodes [9–11] with an overall electrochemical process, similar to that occurring in biological systems, which implies the interconversion of the redox couple RSH/RSSR [12]. Adsorption phenomena and the effect of electrode surface oxides complicate the electrochemical oxidation of Hcy at these electrodes [11]. Chemically modified electrodes have been used to overcome such inconvenience [9–11].

Nanotechnology is nowadays sharing knowledge, tools, techniques, and information on electrochemistry and electroanalysis with other fields [12]. Carbon nanotubes represent one of the commonly used building blocks of nanotechnology. We, therefore, proposed on the basis of previous work [13–20], CHP as a mediator for the rapid, sensitive, and highly selective voltammetric determination of Hcy on the surface of an MWCNTPE. The results showed that the catalytic current depends on the concentration of Hcy. Cyclic voltammetry (CV) and double potential step chronoamperometry are employed to establish the electrocatalytic behavior of CHP. The proposed method is selective, sensitive, and fast for the determination of Hcy in real samples such as serum and urine.

## 2. Experimental

### 2.1. Apparatus and Reagents

All the voltammetric measurements were performed using an Autolab PGSTAT 302N, potentiostat/galvanostat (Utrecht, The Netherlands) connected to a three-electrode cell, Metrohm (Herisau, Switzerland) Model 663 VA stand, linked with a computer (Pentium IV, 1, 200 MHz) and with Autolab software. A platinum wire was used as the auxiliary electrode. MWCNTPE and Ag/AgCl/KCl_sat_ were used as the working and reference electrodes, respectively. The carbon nanotubes were characterized by scanning electron microscopy (SEM) (Seron Tech. AIS 2100). A digital pH/mV-meter (Metrohm model 710) was applied for pH measurements. Spectrally pure graphite powder (particle size <50 *μ*m) from Merck was used as the substrate for the preparation of the carbon paste electrode.

### 2.2. Synthesis of Multiwall Carbon Nanotubes

**Figure 1:**
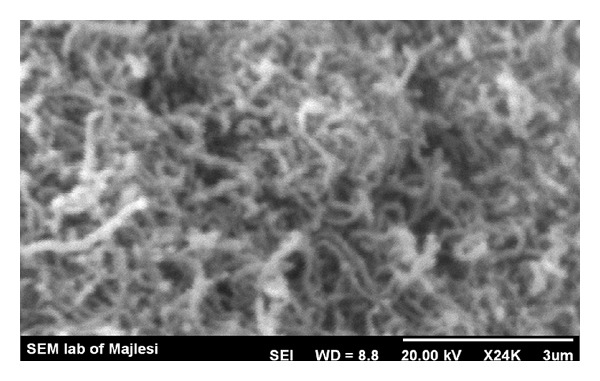
SEM image of MWCNTs.

The nanotubes were grown by chemical vapor deposition. Several transition metal catalysts have been shown to be active for generation of carbon nanotubes [21]. In this work MWCNTs were synthesized from acetylene on a Fe : Co : CaCO_3_ catalyst at 720°C. For the production of carbon nanotubes, approximately 100 mg of catalyst containing 5 wt% Fe-Co with a mole ratio of 1 : 1 was weighed and spread into a thin layer onto a quartz boat positioned horizontally inside of a resistive tube furnace under nitrogen flow. The furnace temperature was then set at the reaction temperature, while accurately controlled. When temperature reached to 720°C, acetylene was introduced at 3.0 mL/min, while the flow of nitrogen maintained at 200 mL/min. After rinsing the system with nitrogen, reaction product was collected from the quartz boat. For purification, raw MWCNT samples were sonicated (40 kHz) in diluted nitric acid (30% HNO_3_) for 30 min, filtered, washed with distilled water to remove acid, and finally dried at 120°C overnight. The residue of as-prepared MWCNTs was placed inside a Pyrex tube and oxidized in a furnace at 350°C in air for different time periods to remove carbon impurities (Figure 1). The diameter, length, purity, and other specifications of synthesized MWCNTs are summarized in Table 1.

### 2.3. Preparation of the Electrode

Graphite powder (0.900 g) was dissolved in diethyl ether and hand mixed with 0.100 g carbon nanotubes in a mortar and pestle. The solvent was evaporated by stirring. A syringe was used to add paraffin to the mixture, which was mixed well for 40 min until a uniformly wetted paste was obtained. The paste was then packed into a glass tube. Electrical contact was made by pushing a copper wire down the glass tube into the back of the mixture. When necessary, a new surface was obtained by pushing an excess of the paste out of the tube and polishing it on a weighing paper.

### 2.4. Preparation of Real Samples

Urine samples were stored in a refrigerator immediately after collection. Ten milliliters of the sample was centrifuged for 5 minutes at 1500 rpm. The supernatant was diluted 100 times with universal buffer pH = 4.0. The solution was transferred into the voltammetric cell to be analyzed without any further pretreatment. Standard addition method was used for the determination of Hcy in real samples.

### 2.5. Optimization of CHP Concentration

The influence of CHP concentration on the peak currents was studied in the concentration range of 50–500 *μ*M CHP at pH 4.0. The results showed that by increasing the CHP concentration up to 400 *μ*M the net peak current increased, whereas further increasing the concentration of CHP caused a decrease in the magnitude of the peak current. Therefore, 400 *μ*M was selected as the optimal CHP concentration.

## 3. Results and Discussion

### 3.1. Electrochemistry of Mediator

 The experimental results showed well-defined and reproducible anodic and cathodic peaks related to CHP_Red_/CHP_Ox_ redox couple with quasireversible behavior and with a peak separation potential of Δ*E*_*p*_ = 110 mV (*E*_*pa*_ − *E*_*pc*_). One of the methods to calculate the electron transfer rate constant (*k* ^0^) for reversible and quasireversible systems was given by Nicholson [22]. This method was based on cyclic voltammetry procedure and potential difference between the peaks (Δ*E*_*p*_). They presented a working curve, *n*Δ*E*_*p*_ versus *ψ*, where *ψ* was defined as follows:

(1) $$\psi =\frac{K_{0}{\left(\text{RT}\right)}^{1/2}}{{\left(nF\pi \nu D_{\text{app}}\right)}^{1/2}},$$

where *D*_app_ is diffusion coefficient of the mediator, *n* is number of electrons, and *ν* is scan rate. Using the previous equation *k*_0_ value was calculated as 4.35 × 10^−3^ cms^−1^.

### 3.2. Catalytic Effect

**Scheme 1:**
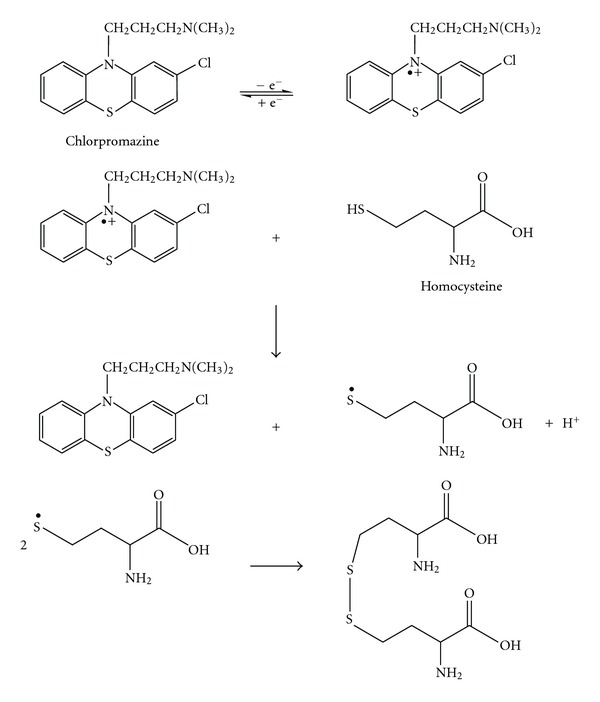
The role of chlorpromazine on the oxidation of Hcy.

Hcy is an oxidisable compound and can be detected by electrochemical methods based on anodic oxidation. The role of CHP as a mediator in the Hcy oxidation at the surface of MWCNTPE is shown in Scheme 1. As shown in Scheme 1, CHP was oxidized directly at the surface of the modified electrode. Then, the oxidized form of CHP reacts with Hcy to oxidize Hcy.

**Figure 2:**
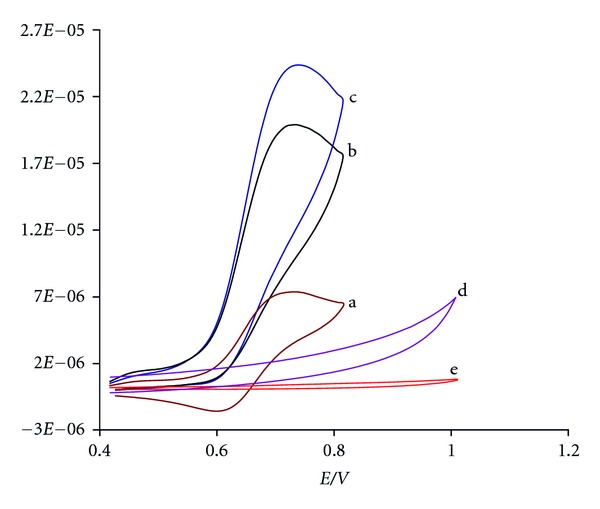
Cyclic voltammograms of (a) 400 *μ*M CHP at the surface of MWCNTPE; (b) 400 *μ*M CHP at the surface of CPE in the presence of 200 *μ*M Hcy; (c) 400 *μ*M CHP at the surface of MWCNTPE in the presence of 200 *μ*M Hcy; (d) 200 *μ*M Hcy at the surface of MWCNTPE; (e) MWCNTPE in the buffer solution. Conditions: 0.04 mol L^−1^ universal buffer (pH 4.0), scan rate of 20 mVs^−1^.

Result shows that, at the surface of an MWCNTPE (without mediator), Hcy could not oxidize until the potential reached +1.0 V (Figure 2 curve d). Also, anodic peak current that is observed for CHP in the absence of Hcy (Figure 2 curve a) increases greatly in 200.0 *μ*M Hcy solution, while the corresponding cathodic peak decreases on the reverse scan (curves c). Therefore, the CHP_(Ox)_ electrogenerated at the MWCNTPE undergoes a catalytic reduction by Hcy back to CHP_(Red)_, which can then be electrochemically reoxidized to produce an enhancement in the anodic current. Therefore, Hcy can be detected in the CHP potential about 0.717 V versus Ag|AgCl|KCl_sat_ at the surface of MWCNTPE. In same condition, when we compared the oxidation of Hcy at the surface of bare carbon paste electrode (CPE) in the presence of mediator (curve b), it was observed that an enhancement of the anodic peak current occurred at MWCNTPE versus the value obtained with CPE. In other words, the data obtained clearly show that the combination of multiwall carbon nanotubes and the mediator definitely improves the characteristics of the electrode for the oxidation of Hcy.

**Figure 3:**
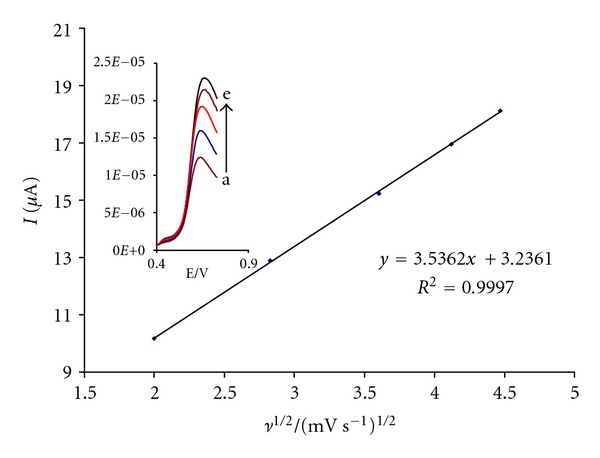
Plot of I_pa_ versus *ν* ^1/2^ for the oxidation of 150 *μ*M Hcy in the presence 400 *μ*M CHP at the surface of MWCNTPE. Inset: linear sweep voltammetry of 150 *μ*M Hcy in the presence 400 *μ*M CHP at various scan rates as (1) 4, (2) 8, (3) 13, (4) 17, and (5) 20 mV s^−1^ in 0.04 M buffer solution (pH 4.0).

The effects of the scan rate on the peak current at the MWCNTPE in the presence of mediator in a 0.04 mol L^−1^ universal buffer solution were investigated in the range of 4.0–20.0 mVs^−1^ by cyclic voltammetry in the presence of 150.0 *μ*M Hcy at pH = 4.0 (Figure 3). The result shows that the peak current increased linearly with the square root of the scan rate (Figure 3), which indicates a diffusion controlled oxidation process occurring at the MWCNTPE in the presence of mediator.

**Figure 4:**
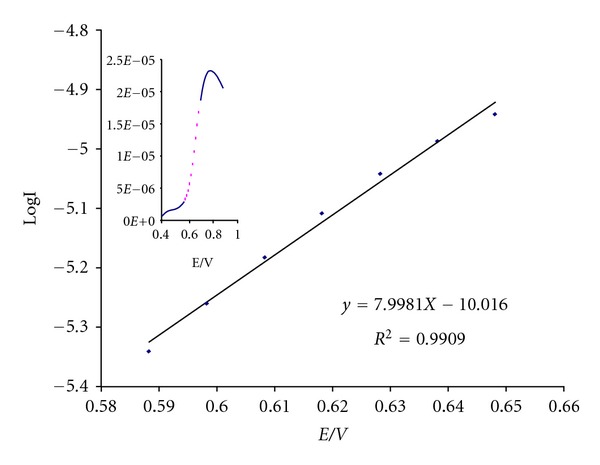
Tafel plot 400 *μ*M CHP at the surface of MWCNTPE in 0.04 mol L^−1^ universal buffer (pH 4.0) at a scan rate of 20 mV s^−1^ in the presence of 150 *μ*M Hcy.

In order to get the information about the rate determining step, a Tafel plot was developed for MWCNTPE in the presence of mediator using the data derived from the raising part of the current-voltage curve (Figure 4). The slope of the Tafel plot is equal to *n*(1 − *α*)*F*/2.3RT or to 7.9981 decade V^−1^. Using these data gives *n*_*α*_ = 0.53. If *n* = 1, then *α* = 0.53. This value of *α* indicates that the activation free energy curve is symmetrical in this system.

**Figure 5:**
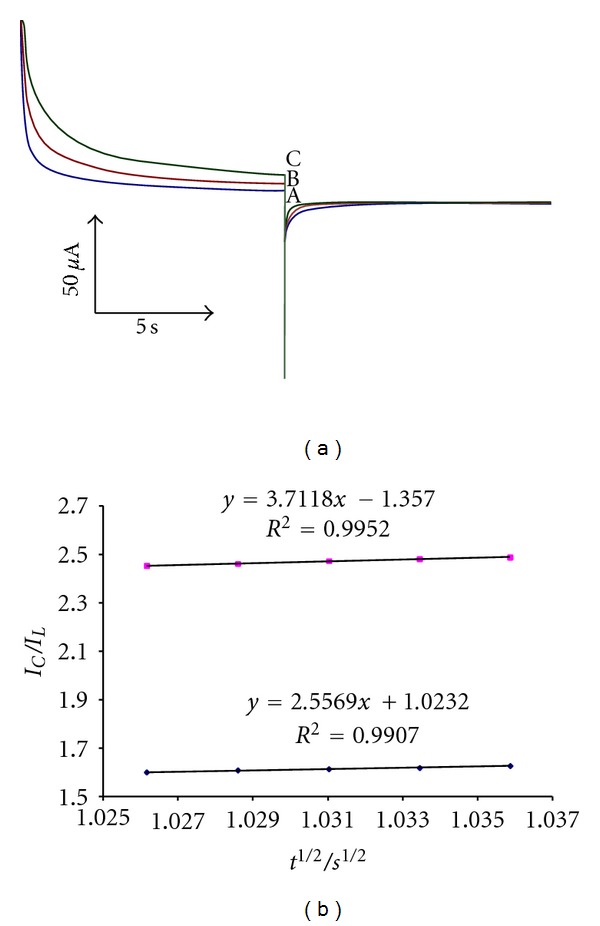
(a) Chronoamperograms obtained at the MMWCNTPE in the absence (A) and in the presence of (B) 250 and (C) 500 *μ*M Hcy in a buffer solution (pH 4.0). (b) dependence of *I*_*C*_/*I*_*L*_ on the *t* ^1/2^ derived from the chronoamperogram data.

In order to obtain an estimation of the rate constant of the catalytic oxidation (*k*_*h*_′) of Hcy, chronoamperometric method was applied to the system (Figure 5(a)). The rate constant for the chemical reaction between CHP and Hcy (*k*_*h*_) is determined according to the method of Galus [23]:

(2) $$\frac{I_{C}}{I_{L}}=\pi^{1/2}\gamma^{1/2}=\pi^{1/2}{\left(k_{h}t\right)}^{1/2},$$

where *I*_*C*_ is the catalytic current of CHP in the presence of Hcy and *I*_*L*_ is the limiting current in the absence of Hcy. From the slope of *I*_*C*_/*I*_*L*_ versus *t* ^1/2^ for five different concentrations of Hcy, the average value of *k*_*h*_ was calculated to be 8.551 × 10^3^ M^−1^ sec^−1^ (Figure 5(b)). This value of rate constant explains the sharp catalytic peak observed for the oxidation of Hcy at the surface of MWCNTPE in the presence of mediator.

### 3.3. Dynamic Range and Limit of Detection

Square wave voltammetry (SWV) was used for determination of Hcy. The SW voltammograms clearly show that the plot of peak current versus Hcy concentration is linear for 0.1–210 *μ*M of Hcy, the regression equation being *I*_*p*_(*μ*A) = (0.0680 ± 0.0012)*C*_Hcy_ + (21.7900 ± 0.0631) (*r* ^2^ = 0.9900, *n* = 14), where *C* is *μ*M concentration of Hcy and *I*_*p*_ is the peak current. The detection limit was determined as 0.08 *μ*M Hcy according to the definition of *Y*_LOD_ = *Y*_*B*_ + 3*σ*. This value of detection limit, the linear dynamic range, and the sensitivity for Hcy observed for the MWCNTPE in the presence of mediator are comparable and even better than those obtained for several other modified electrodes (Table 2).

### 3.4. Interference Study

In order to evaluate the selectivity of the proposed method in Hcy determination, the effects of various foreign species on the determination of 5.0 *μ*M Hcy were investigated. The tolerance limit was taken as the maximum concentration of the foreign substances causing an approximately ±5% relative error in the determination. The results are given in Table 3. Although ascorbic acid typically shows some interference, it could be minimized if necessary by using ascorbic oxidase enzyme which exhibits a high selectivity to the oxidation of ascorbic acid.

### 3.5. Determination of Hcy in Real Samples

In order to demonstrate the electrocatalytic oxidation of Hcy in real samples, we examined the voltammetric determination of Hcy in serum and urine samples. The results were compared with those obtained from the published method used for Hcy determination. The results, reported in Table 4, demonstrate the ability of CHP as a suitable homogeneous mediator at a surface of MWCNTPE for voltammetric determination of Hcy with a high selectivity and a good reproducibility.

## 4. Conclusion

This work describes the ability of the multiwall carbon nanotube paste electrode in the presence of CHP as a suitable mediator for catalytic determination of Hcy. It has been found that with cyclic voltammetry the oxidation of Hcy occurs at a potential of about +0.717 V on the surface of the MWCNTPE in the presence of CHP, while the oxidation of Hcy does not take place at the surface of a carbon nanotube paste electrode without CHP up to +1.0 V. The proposed method is sensitive to Hcy levels as low as 0.08 *μ*M. The kinetic parameter of the electrocatalytic process and the diffusion coefficients of Hcy in an aqueous solution was determined. Finally, this method was also examined as a selective, simple, and precise electrochemical sensor for the determination of Hcy in real samples such as serum and urine.

## Figures and Tables

**Table 1:** Specification of synthesised multiwalled carbon nanotubes by chemical vapor deposition method.

Catalyst	Co : Fe
Color	Black
Purity	>95%
Outside diameter (OD)	70–100 nm
Inside diameter (ID)	50–70 nm
Length	10–50 *μ*m
Special surface area (SSA)	235 m^2^/g
Bulk density	0.07 g/cm^3^
True density	~2.1 g/cm^3^

**Table 2:** Comparison of the efficiency of some electrochemical methods in the determination of Hcy.

Electrode	Methods	LOD (*μ*mol L^−1^)	LDR (*μ*mol L^−1^)	Reference
Carbon paste	HPLC with amperometric detection	0.03	0.1–5	[9]
Glassy carbon	Amperometry	0.06	0.1–60.0	[10]
Carbon-nanotube paste	Amperometry	4.6	5.0–200.0	[11]
Carbon-nanotube paste	SWV	0.08	0.1–210.0	This work

**Table 3:** Interference study for the determination of 5.0 *μ*mol L^−1^ Hcy under the optimized conditions.

Species	Tolerance limits (*m*_substance_/*m*_Hcy_)
Glucose, fructose, lactose, sucrose	800
Li^+^, Cl^−^, folic acid, histidine, alanine, phenyl alanine, methionine, glycine, methanol, ethanol, urea, SCN^−^, SO_4_^2−^	600
Starch	Saturation
Ascorbic acid	5

**Table 4:** Concentration values obtained from the proposed and published methods for Hcy analysis in real samples.

Sample	Added (*μ*M)	Expected (*μ*M)	Proposed method (*μ*mol L^−1^)	Published method (*μ*mol L^−1^)	*F*_ex_	*F*_tab_, (0.05);2,2	*t*_ex_	*t*_tab_ (98%)
Urine	—	—	Less than limit of detection	Less than limit of detection	—	—	—	—
	5.00	5.00	4.85 ± 0.31	5.42 ± 0.51	5.8	19	2.3	3.8
	5.00	10.00	10.21 ± 0.44	10.48 ± 0.60	6.2	19	2.5	3.8
	20.00	30.00	30.12 ± 0.25	30.33 ± 0.41	5	19	2.0	3.8
Serum			Less than limit of detection	Less than limit of detection	—	—	—	—
6	50.00	50.00	50.63 ± 0.65	50.87 ± 0.92	8.6	19	3.5	3.8
7	20.00	70.00	70.55 ± 0.58	70.67 ± 0.75	7.0	19	3.0	3.8

*F*_ex_: calculated *F* value; reported *F* value from *F*-test table with 95% confidence level and 2/2 degree of freedom; *t*_ex_: calculated *t*; *t*_tab_ (98%): reported *t* value from student's *t*-test table with 98% confidence level.
